# Overcoming single model bias through GRACE and multi model data reveals Iran water storage depletion drivers

**DOI:** 10.1038/s41598-026-51961-6

**Published:** 2026-05-15

**Authors:** Xilong Yuan, Fengwei Wang, Yunqi Zhou, Shijian Zhou

**Affiliations:** 1 School of Surveying and Geoinformation Engineering, East China University of Technology, Nanchang, 330013 China; 2 College of Surveying and Geo-informatics, Tongji University, Shanghai, China; 3 School of Geographical Sciences, University of Bristol, Bristol, BS8 1SS UK; 4 School of Software, Nanchang Hangkong University, Nanchang, 330063 China

**Keywords:** GRACE, Hydrological models, TWSA, Least-squares method, Linear trend, Climate sciences, Environmental sciences, Hydrology, Water resources

## Abstract

Rapid changes in terrestrial water storage (TWS) pose serious threats to water security in arid and semi-arid regions such as Iran. However, the inherent uncertainties of individual hydrological models hinder robust assessments of the respective impacts of natural variability and anthropogenic influence on water storage dynamics in these areas. To address this issue, our study integrates GRACE/GRACE-FO satellite gravity data, five mainstream hydrological models (GLDAS-Noah, GLDAS-VIC, GLDAS-CLSM, ERA5, and WGHM), and GPM global precipitation data. Four observational datasets related to precipitation, runoff, and evapotranspiration were derived, and 64 different hydrological model combinations were constructed. These combinations were comprehensively evaluated against Mascon products as a benchmark. Ultimately, the model combination with the best fitting performance was selected for spatial and temporal variation analysis and attribution analysis. The findings reveal that: (1) The model combination constructed using ERA5-derived evapotranspiration and runoff data, combined with precipitation data from VIC/CLSM, exhibits the highest consistency with Mascon data. (2) In densely populated northern and southwestern regions, the natural water flux shows significant upward trends. Nevertheless, TWSA declines there because anthropogenic extraction (captured in the net residual) outweighs the natural increase, causing groundwater discharge to surface systems and amplifying evapotranspiration and runoff losses. (3) In sparsely populated arid central regions, TWSA remains relatively stable, with an average annual natural water anomaly change rate of approximately + 0.01 cm/yr, primarily due to the offsetting effects of precipitation and evapotranspiration. This study systematically evaluates the applicability of a multi-model approach in arid regions. The integrated hydrological modeling framework developed herein provides a methodology for selecting model configurations and quantifying associated uncertainties in water storage assessment, thereby offering a scientific basis for sustainable water resource management in arid environments.

## Introduction

The Gravity Recovery and Climate Experiment (GRACE) and its successor mission (GRACE-FO) provide a direct and independent approach for monitoring terrestrial water storage anomalies (TWSA) at global and regional scales by detecting temporal variations in the Earth’s gravity field. Data from these missions have proven critical for global water resource assessment^1^, monitoring and early warning of extreme hydrological events^2^, and understanding the evolution of the water cycle under global climate change^3^. However, GRACE/GRACE-FO observations represent the integrated change in total terrestrial water storage, encompassing soil moisture, groundwater, snow water equivalent, surface water, and biomass, without directly resolving individual components. To decipher the sources of TWSA signals, integrating GRACE observations with hydrological models is essential.

Since the introduction of the first primitive land surface model by Manabe^4^, a wide array of hydrological models has been developed for TWSA analysis. Contemporary research primarily relies on four categories: (1) Land Surface Models (LSMs)^5^, (2) Global Hydrological Models (GHMs)^6^, (3) Semi-distributed and Distributed Physical Models^7,8^, and (4) Data Assimilation and Fusion Models^9^. Among LSMs, the Global Land Data Assimilation System (GLDAS)^10^ and ERA5-Land^11^ are the most widely used data products, which provide global coverage, long-term time series, and output numerous energy and hydrological variables. However, their simplified parameterizations of hydrological processes and general lack of anthropogenic forcing often lead to poor simulation performance in regions intensely impacted by human activities^12^. A prominent example of GHMs is the WaterGAP Global Hydrology Model (WGHM)^13^, which delivers a comprehensive simulation of the global water cycle. Nevertheless, uncertainties in its globally calibrated parameters can result in biased performance at regional scales^14^. Semi-distributed and distributed physical models are often used to investigate the impact of model structure on simulation accuracy by comparing different configurations. Distributed models (e.g., SWAT, SHE) can capture the spatiotemporal variability of hydrological processes more finely, while semi-distributed models (e.g., TOPMODEL) offer a better balance between computational efficiency and acceptable accuracy. However, these models typically require extensive spatial input data, and their numerous parameters can introduce significant uncertainty^15^. Data assimilation and fusion models represent a cutting-edge approach and a mainstream research direction for enhancing the accuracy of TWSA studies. Their core principle is to fuse observational data (like GRACE) with hydrological model simulations to generate datasets that are closer to reality and spatiotemporally consistent^16^. GLDAS itself is a representative land surface data assimilation system. Current research focuses on developing more advanced fusion techniques, such as using Independent Component Analysis^17^ to extract distinct spatial features representing different hydrological processes from high-resolution models and recombining them under the constraints of GRACE observations. The integration of deep learning with temporal assimilation frameworks also offers novel avenues, for instance, in constructing global 320-meter resolution surface soil moisture maps using deep learning models. Compared against data from approximately 1000 global in-situ sensors, such models significantly enhance spatial resolution while maintaining accuracy^18^. However, these methods are often complex, computationally intensive, face challenges in estimating error covariances between different observations and models, and deep learning, in particular, struggles with insufficient physical interpretability^19^. In summary, while existing models offer distinct advantages for supporting TWSA research, they also possess inherent limitations. Therefore, systematically evaluating and selecting the most suitable model for a specific study area is crucial for enhancing the accuracy and reliability of TWSA analysis, particularly in arid regions significantly impacted by human activities, such as the Middle East.

To investigate the severe water resource crisis in arid regions, this study selects Iran as a representative case. Iran is a typical arid to semi-arid country in the Middle East, where water resources are under severe stress due to the dual pressures of climatic aridity and intensive human activities. The region is experiencing severe and persistent losses in both surface and groundwater storage, a common challenge faced by many arid regions worldwide. Therefore, an in-depth investigation of Iran provides a reference for understanding and addressing similar water crises in other comparable regions. Existing research indicates that the region is experiencing severe terrestrial water storage depletion, with a reported negative trend of approximately − 25 ± 3 Gt/yr in the central western region^20^. Furthermore, groundwater storage in its central plateau basins has been declining at a rate of 11.55 mm/yr due to unsustainable extraction^21^. In this process, abstracted groundwater is transferred to the surface; only a minor fraction may return to the aquifer via recharge, while the majority is subject to loss through evapotranspiration and runoff. Consequently, this irreversible transfer and loss of subsurface water directly contributes to the observed decline in TWSA in the region. However, the performance and uncertainty of any single hydrological model make it difficult to conduct a systematic assessment of TWSA in this region, limiting our ability to accurately quantify water storage changes and understand their driving mechanisms. Consequently, this study establishes a systematic multi-model comparison framework to investigate the relative strengths and weaknesses of different data sources in simulating TWSA over Iran. We selected five representative hydrological models, including three from the Global Land Data Assimilation System (GLDAS: Noah, VIC, CLSM), along with ERA5 and WGHM, and GPM global precipitation data^22^. This ensemble was chosen based on comprehensiveness, widespread application, and complementary strengths. The GLDAS models are well-established and provide a coherent suite with differing physical parameterizations, facilitating an assessment of structural uncertainty. ERA5-Land offers high-resolution, globally consistent land surface fields from advanced data assimilation. WGHM explicitly integrates human water use, which is critical for representing anthropogenic impacts in arid regions like Iran. Collectively, these widely used and publicly available models encompass a range of key hydrological processes, enabling a robust multi-model evaluation of water storage dynamics. From these, we derived four distinct datasets for precipitation, four for evapotranspiration, and four for runoff. Based on these datasets, we constructed a total of 128 hydrological model combinations across two spatial resolutions. These combinations were comprehensively evaluated against a benchmark formed by the average of three Mascon solutions, aiming to identify the “optimal combined model” that most reliably reproduces the spatiotemporal evolution of TWSA over Iran. This optimal model was then used for an in-depth analysis of spatiotemporal dynamics and causal diagnosis. Our findings reveal substantial differences in the performance of various models in assessing Iranian TWSA. Specifically, ERA5-derived evapotranspiration and runoff data demonstrated superior performance, as did precipitation data from VIC/CLSM, whereas the Noah model generally underestimated TWSA trends. Spatiotemporal analysis indicates a stronger human impact on TWSA in densely populated areas, where evapotranspiration and runoff are the primary factors contributing to the terrestrial water storage decline. In contrast, the arid central region exhibited relative stability in TWSA, attributable to lower population density and the counterbalancing effects of precipitation and evapotranspiration. This study establishes a multi-model, multi-dataset framework for screening an “optimal combination.” By comparing 128 different combinations constructed from five major hydrological models and GPM precipitation data, we identified the model system with the best simulation performance for Iranian TWSA, thereby revealing performance disparities among the models. Based on this optimal model, we analyzed the causes of TWSA changes in Iran. This approach enhances the accuracy of TWSA monitoring in complex environments and deepens the scientific understanding of water cycle processes and their driving mechanisms in arid regions.

## Datasets and study regions

### Datasets

#### GRACE data

This study employed the Release 06 (RL06) mascon solutions from the Center for Space Research (CSR) at the University of Texas at Austin, and from the National Aeronautics and Space Administration’s (NASA) Jet Propulsion Laboratory (JPL) and Goddard Space Flight Center (GSFC). The data span the period from April 2002 to December 2023, covering observations from both the GRACE mission and its successor, GRACE-FO. These mascon solutions provide higher spatial resolution, which effectively reduces stripe errors^23^, incorporates degree-1 terms, and uses a replacement for the C₂₀ coefficient. The Glacial Isostatic Adjustment (GIA) effect was corrected using the ICE6G_D model^24^. Since the JPL and GSFC mascons have a spatial resolution of 0.5° compared to the higher 0.25° resolution of the CSR product, the CSR data were upscaled to 0.5° using a latitude-cosine-based area-weighting method to ensure spatial consistency for subsequent comparative analysis.

#### Hydrological models

This study aims to analyze changes in the hydrological cycle from April 2002 to December 2023 through a multi-model integration approach. Five hydrological models were employed: the Noah, CLSM, and VIC models within the GLDAS framework, the ERA5 reanalysis dataset from the European Centre for Medium-Range Weather Forecasts (ECMWF), and the WGHM from the University of Kassel, Germany. GLDAS, jointly developed by NASA GSFC and the National Centers for Environmental Prediction (NCEP), drives its constituent land surface models by assimilating various satellite and ground-based observations. This study uses GLDAS Version 2.1 data. The spatial resolution is 0.25° for the Noah model and 1.0° for both the CLSM and VIC models, all with a monthly temporal resolution. Data for precipitation, evapotranspiration, surface runoff, and subsurface runoff were uniformly extracted from these models. A key advantage of the GLDAS ensemble is its operation under a unified forcing framework, providing an ideal basis for multi-model comparison and uncertainty assessment. Specifically, the Noah model is structurally robust and widely adopted, demonstrating good performance in simulating soil temperature and moisture^25^. The CLSM, based on a topographic catchment concept, offers a more physically-based representation of groundwater-soil water interactions^26^. The VIC model excels in runoff simulation, particularly in capturing extreme hydrological events, owing to its variable infiltration capacity scheme^27^. ERA5, the fifth-generation atmospheric reanalysis from ECMWF, was used in its monthly-averaged form with a spatial resolution of 0.25°. Precipitation, evapotranspiration, and runoff data were extracted. A significant strength of ERA5 is its advanced four-dimensional variational data assimilation system, which integrates vast observational datasets to produce a spatiotemporally complete and physically consistent product. However, biases may persist for variables like precipitation in data-sparse regions and complex terrain. WGHM is a comprehensive global model designed to quantify human impacts on the water cycle. With a spatial resolution of 0.5° and a monthly temporal resolution, its simulated anthropogenic water use impacts (e.g., agricultural irrigation, industrial and domestic withdrawals) were used in this study for comparison with the natural simulations from other models, highlighting its unique value. Its simulation accuracy, however, is highly dependent on the accuracy and completeness of the underlying human water use data. By integrating these five models, each with distinct strengths in physical mechanisms, input data, and focus areas, this study seeks to provide a more comprehensive revelation of both natural variability and anthropogenic influences on hydrological processes, thereby enhancing the robustness of the findings. The resolution and temporal coverage of the different models are summarized in Table 1.

**Table 1:** Description of the hydrological models, including their temporal coverage and spatial resolution.

Model	Period	Spatial resolution	Data access
GLDAS-Noah	2002.4–2023.12.4.12	0.25° × 0.25°	https://ldas.gsfc.nasa.gov/gldas
GLDAS-CLSM	2002.4–2023.12.4.12	1° × 1°	https://ldas.gsfc.nasa.gov/gldas
GLDAS-VIC	2002.4–2023.12.4.12	1° × 1°	https://ldas.gsfc.nasa.gov/gldas
ERA5-Land	2002.4–2023.12.4.12	0.25° × 0.25°	https://www.ecmwf.int/en/forecasts/datasets/reanalysis-datasets/era5
WGHM	2002.4–2019.12.4.12	0.5° × 0.5°	https://www.uni-frankfurt.de/45218023/WaterGAP

#### Global precipitation data

Given that the CLSM and VIC models within the GLDAS framework share a unified precipitation forcing source, the Global Precipitation Measurement (GPM) mission data were incorporated to provide more reliable precipitation inputs. GPM significantly improves global precipitation retrieval accuracy through its core observatory’s Dual-frequency Precipitation Radar (DPR)^28^ and GPM Microwave Imager (GMI)^29^, utilizing multi-source data fusion algorithms. The specific product used herein is the Level-3 monthly mean dataset, which boasts a high spatial resolution of 0.1°, serving as a high-quality, independent precipitation benchmark for hydrological modeling.

### Study area

**Fig. 1:**
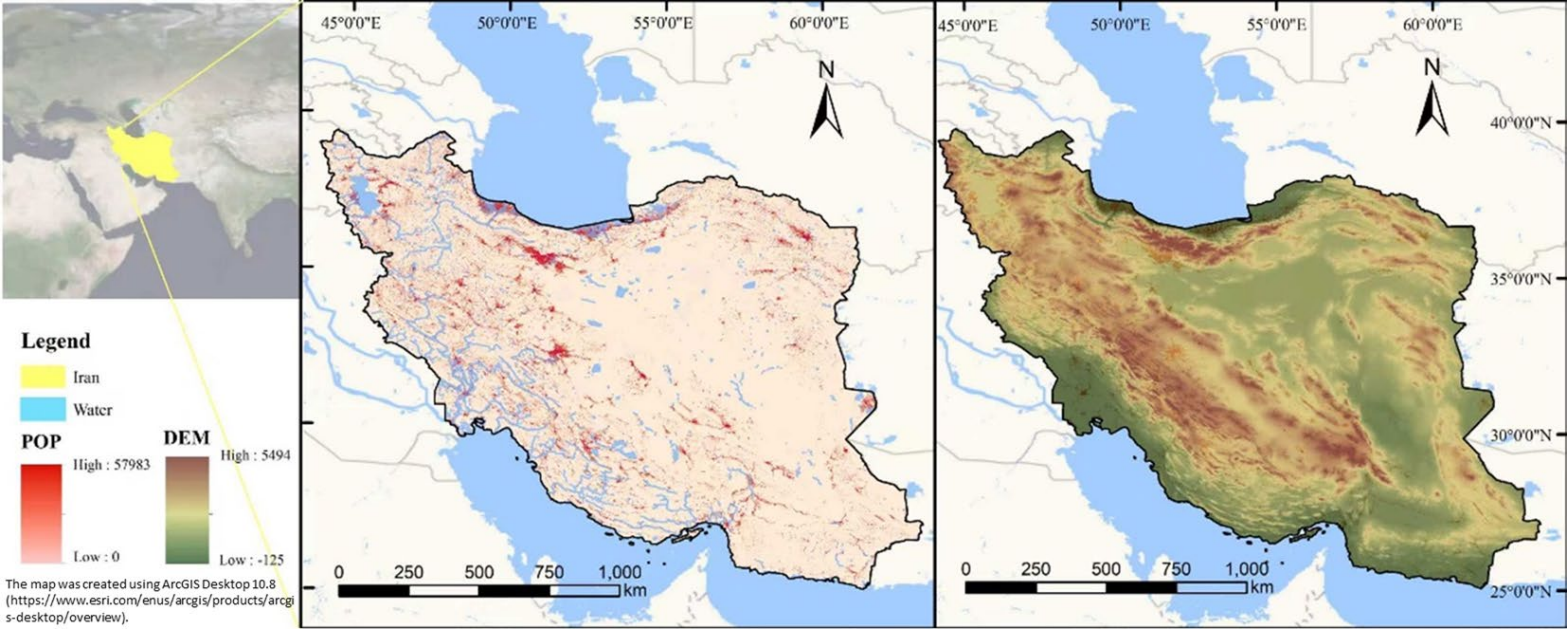
Location map of the study area. Left: Population distribution and river network as of 2023; Right: DEM.

The study area encompasses the Islamic Republic of Iran (Iran), located in southwestern Asia at the transitional zone between Western Asia and Central Asia (Fig. 1). It extends from 44°E to 64°E in longitude and 24°N to 40°N in latitude, covering a total area of 1.64 million km². The topography is fundamentally controlled by the Zagros Fold and Thrust Belt, which defines a series of NW-SE trending mountain ranges. This complex topography governs a pronounced climatic gradient. Conditions range from the humid temperate climate of the Caspian Highlands, through the hyper-arid deserts of the central plateau, to the hot and humid coastal plains along the Persian Gulf. These climatic and topographic constraints collectively result in extreme hydrological heterogeneity. According to the Catalogue of Hydrological Analyses for Asia and the Pacific, over 70% of the surface runoff originates in the western mountains, a region that comprises only 25% of the national territory. Furthermore, groundwater is a critical resource, sustaining 55% of agricultural activities and accounting for 92% of total freshwater consumption. Figure 1 shows a location map of the study area, including the population distribution, major river networks, and topography (DEM) of Iran.

## Methodology

**Fig. 2:**
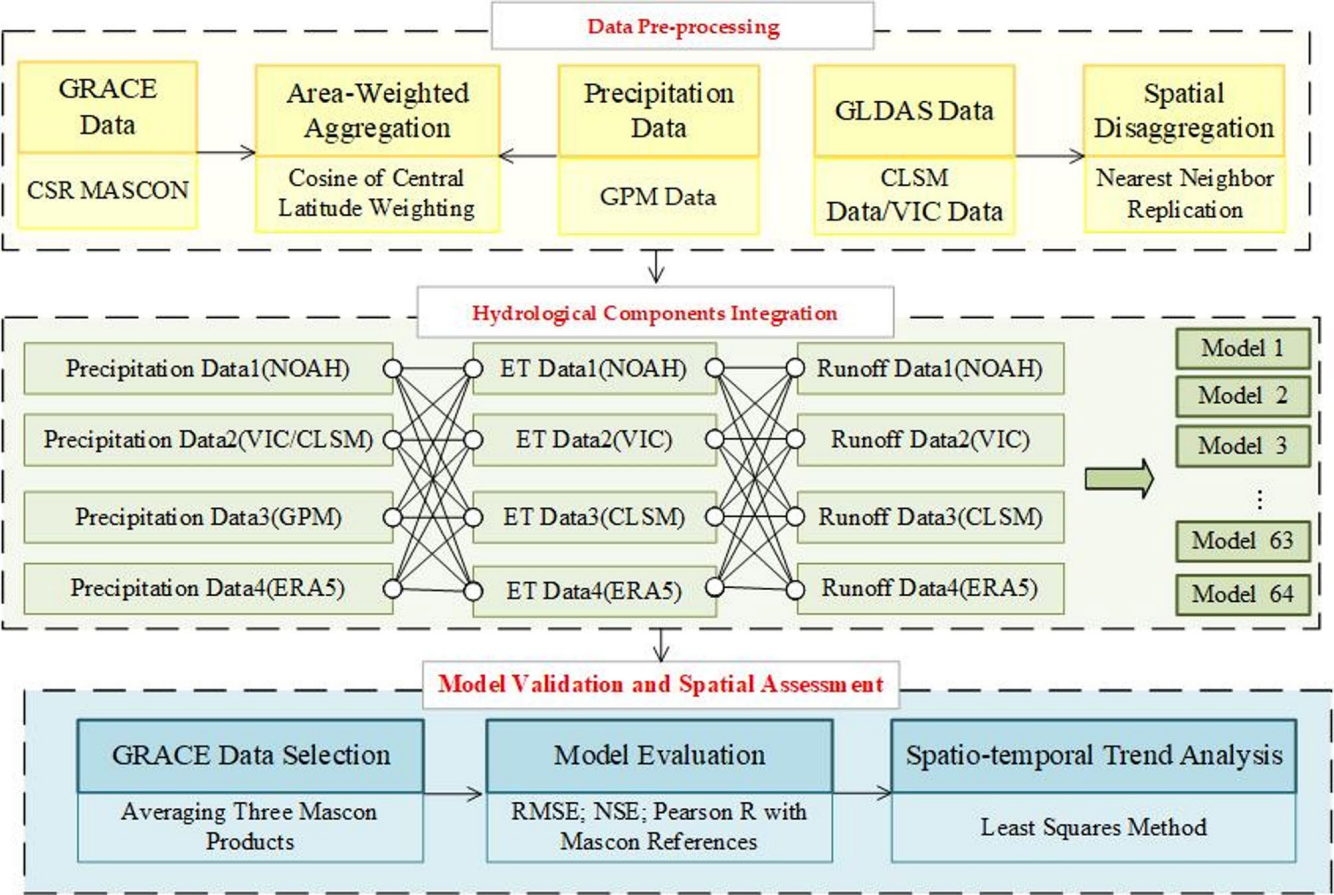
Methodological workflow.

The methodological workflow of this study is illustrated in Fig. 2.

To ensure spatial consistency, datasets were first resampled to common resolutions. The 0.25° CSR Mascon product was upscaled to 0.5° to match the resolution of the JPL and GSFC solutions. Similarly, the 0.1° GPM global precipitation data were upscaled to 0.25° to align with the GLDAS-Noah model. This upscaling was performed using a latitude-cosine area-weighting method^30^, which accounts for the variation in grid cell area due to Earth’s curvature, thereby ensuring the conservation of physical quantities during the transformation. The high-resolution data were first mapped onto the target coarser grid using Eq. (1):

1 $$\:\begin{array}{c}f:\left(x,y\right)\to\:\left(\lfloor \frac{x-{X}_{min}}{i}\rfloor,\lfloor \frac{y-{Y}_{min}}{i}\rfloor \right)\end{array}$$

where $\:x$, $\:y$ are the original grid coordinates, $\:X$, $\:Y$ are the output grid indices, and $\:i$ is the target spatial resolution.

Subsequently, the mapped values were aggregated using a cosine-of-latitude area weight, as defined in Eq. (2):

2 $$\:\begin{array}{c}{D}_{low}\left(X,Y,t\right)=\frac{\sum\:_{(x,y)\in\:{\varOmega\:}_{X,Y}}\:cos\left({\phi\:}_{Y}\right)\cdot\:{D}_{high}\cdot\:(x,y,t)\cdot\:I(x,y,t)}{\sum\:_{(x,y)\in\:{\varOmega\:}_{X,Y}}\:I(x,y,t)}\end{array}$$

where $\:t$ is time, $\:{D}_{low}\left(X,Y,t\right)$ is the upscaled data, $\:{D}_{high}\cdot\:(x,y,t)$ is the original high-resolution data, $\:{\phi\:}_{Y}$ is the central latitude of the coarse grid cell, and $\:I(x,y,t)$ is a binary indicator function for valid data.

Conversely, the 1.0° resolution data from GLDAS-VIC and GLDAS-CLSM were downscaled to 0.25° to match GLDAS-Noah, employing a nearest-neighbor replication method described by Eq. (3):

3 $$\:\begin{array}{c}{D}_{high}\left(X,Y,t\right)={D}_{low}\left(\lceil\frac{x}{r}\rceil,\lceil\frac{y}{r}\rceil,t\right)\end{array}$$

where $\:r$ is the resolution refinement factor.

Following the spatial harmonization via Eqs. (1)–(3), a background gravity field was established from the monthly averages over January 2004–December 2009; this field was subsequently subtracted from all datasets as the reference gravity field. Monthly total precipitation (P), evapotranspiration (ET), and runoff (RUNOFF) were then extracted from each hydrological model using the latitude-cosine area-weighting method. Furthermore, given that nearest-neighbor resampling may introduce spatial artifacts when upscaling, the above procedures were repeated to resample all hydrological models to a 1° spatial resolution grid and re-extract the monthly P, ET, and RUNOFF fields. Finally, by systematically combining the four precipitation, four ET, and four runoff datasets at both spatial resolutions, the terrestrial water storage anomaly (TWSA) for Iran was computed. TWSA was derived from the water balance equation^31^, given by Eq. (4):

4 $$\:\begin{array}{c}\Delta\:TWSA=\Delta P-\Delta ET-\Delta RUNOFF+\Delta Q\end{array}$$

where $\:Q$ represents anthropogenic influences, $\Delta$ represents the linear rate of change of each variable over time.

Given the difficulties in quantifying anthropogenic influences (ΔQ) and their lack of a clear spatiotemporal pattern, this term was neglected in the comparative analysis between the hydrological models and the Mascon benchmark. Based on Eq. (4), the systematic combination of all input data resulted in a total of 128 distinct hydrological model-based TWSA time series. Concurrently, the three Mascon solutions were processed by removing the same background gravity field (derived from the monthly mean over January 2004 to December 2009) and applying a latitude-cosine weighted average to generate a corresponding TWSA time series for each. An ensemble mean TWSA time series was then computed from these three Mascon solutions to serve as the benchmark. Finally, the Root Mean Square Error (RMSE)^32^, Pearson correlation coefficient (R)^33^, and Nash-Sutcliffe Efficiency coefficient (NSE)^34^ were calculated between each of the 128 hydrological model TWSA series and the benchmark Mascon ensemble mean. These metrics were used to evaluate the temporal consistency between the models and the observations. The formulas for RMSE, R, and NSE are given by Eqs. (5)–(7):

5 $$\:\begin{array}{c}RMSE=\sqrt{\frac{1}{n}\sum\limits_{i=1}^{n}\:{\left({M}_{i}-{O}_{i}\right)}^{2}}\end{array}$$

6 $$\:\begin{array}{c}R=\frac{\sum\:_{i=1}^{n}\:\left({M}_{i}-\stackrel{-}{M}\right)\left({O}_{i}-\stackrel{-}{O}\right)}{\sqrt{\sum\:_{i=1}^{n}\:{\left({M}_{i}-\stackrel{-}{M}\right)}^{2}}\sqrt{\sum\:_{i=1}^{n}\:{\left({O}_{i}-\stackrel{-}{O}\right)}^{2}}}\end{array}$$

7 $$\:\begin{array}{c}NSE=1-\frac{\sum\:_{i=1}^{n}\:{\left({M}_{i}-{O}_{i}\right)}^{2}}{\sum\:_{i=1}^{n}\:{\left({O}_{i}-\stackrel{-}{O}\right)}^{2}}\end{array}$$

where $\:{M}_{i}$ is the model-simulated TWSA value at time $\:i$, $\:{O}_{i}$ is the corresponding benchmark Mascon TWSA value at time $\:i$, $\:\stackrel{-}{M}$ and $\:\stackrel{-}{O}$ are their respective means over the $\:n$ time steps.

Using these metrics, principal component analysis (PCA) was applied to comprehensively evaluate all hydrological model combinations at each spatial resolution, from which the optimal model component was selected according to the composite score defined in Eqs. (8), (9):

PCA‑derived weights for the three evaluation metrics were computed using Eq. (8)

8 $$\:\begin{array}{c}{w}_{j}=\frac{\mid\:{l}_{j1}\mid\:}{\sum\:_{k=1}^{m}\:{\mid\:{l}_{k1}\mid\:}^{\:}}\end{array}$$

where $\:\:{l}_{1}={ \lceil {l}_{11},{l}_{21},{l}_{31}\rceil}^{T}$ is the loading vector of the first principal component.

9 $$\:\begin{array}{c}{Score}_{i}=\sum\limits_{j=1}^{m}\:{w}_{j}\times\:{z}_{ij}\end{array}$$

where $\:{Score}_{i}$ denotes the composite score of the $\:i$th hydrological model, $\:m$ =3 is the number of evaluation metrics, $\:{z}_{ij}$ represents the value of the $\:j$th metric for the$\:\:i$th model after positive transformation and min‑max normalization,$\:\:{w}_{j}$ is the weight of the$\:\:j$th metric, derived from the normalized loadings of the first principal component in principal component analysis.

Subsequently, the TWSA time series from this optimal model and the Mascon data were decomposed using a least-squares fitting procedure^35^ to extract the linear trend, annual amplitude, and semi-annual amplitude. The least-squares fitting is defined in Eq. (10):

10 $$\:\begin{array}{c}f=a+bt+{A}_{1}{cos}({\omega\:}_{1}t+{\varphi\:}_{1})+{A}_{2}{sin}({\omega\:}_{2}t+{\varphi\:}_{2})+\epsilon\end{array}$$

where $\:f$ represents the TWSA, $\:a+bt$ is the linear trend, $\:{A}_{1}{cos}({\omega\:}_{1}t+{\varphi\:}_{1})$ represents the annual signal, $\:{A}_{2}s{in}({\omega\:}_{2}t+{\varphi\:}_{2})$represents the semi-annual signal, and $\:\epsilon\:$ is the residual.

Using Eq. (10), the spatial distribution of the linear trend for the optimal model’s TWSA and its individual components ($\:P$,$\:\:ET$, $\:RUNOFF$) was calculated. The anthropogenic influence on TWSA ($\:Q$) for Iran was then derived by rearranging Eq. (4). This derived anthropogenic influence signal was validated against the corresponding component simulated by the WGHM.

To statistically evaluate the significance of the derived linear trends in TWSA and its components, the modified Mann‑Kendall trend test that accounts for autocorrelation^36^ was employed. This method is widely used in hydrological and climatological studies to detect monotonic trends in time series data while being robust against non‑normal distributions and outliers. However, standard Mann‑Kendall tests assume temporal independence; monthly hydrological and GRACE data often exhibit significant positive autocorrelation, which inflates the effective sample size and leads to over‑rejection of the null hypothesis (i.e., artificially low p‑values). To overcome this issue, the modified test corrects the variance of the test statistic using the significant autocorrelation coefficients of the ranks of the detrended series. The modified Mann‑Kendall test statistic $\:S$ and the standardized test statistic $\:Z$ are calculated as follows:

11 $$\:\begin{array}{c}S=\sum\limits_{i=1}^{n-1}\:\sum\limits_{j=i+1}^{n}\:sgn\left({x}_{j}-{x}_{i}\right)\end{array}$$

where $\:S$ is the sum of the sign function applied to all data pairs ($\:{x}_{i}$,$\:{x}_{j}$) for which $\:i$ <$\:\:j$.

12 $$\:\begin{array}{c}sgn\left({x}_{j}-{x}_{i}\right)=\left\{\begin{array}{c}+1,\hspace{0.25em}\hspace{0.25em}\hspace{0.25em}\hspace{0.25em}if({x}_{j}-{x}_{i})>0\\\:0,\hspace{0.25em}\hspace{0.25em}\hspace{0.25em}\hspace{0.25em}if({x}_{j}-{x}_{i})=0\\\:-1,\hspace{0.25em}\hspace{0.25em}\hspace{0.25em}\hspace{0.25em}if({x}_{j}-{x}_{i})<0\hspace{0.25em}\hspace{0.25em}\hspace{0.25em}\hspace{0.25em}\end{array}\right.\end{array}$$

A positive value indicates that $\:{x}_{j}$ is greater than $\:{x}_{i}$, a negative value indicates a downward trend, and a value of zero indicates equality.

13 $$\:\begin{array}{c}\begin{array}{c}Z=\left\{\begin{array}{ccc}\frac{S-1}{\sqrt{{\mathrm{V}\mathrm{a}\mathrm{r}}^{*}\left(S\right)}},&\:S>0&\:\\\:0,&\:S=0&\:\\\:&\:&\:\\\:\frac{S+1}{\sqrt{{\mathrm{V}\mathrm{a}\mathrm{r}}^{*}\left(S\right)}},&\:S<0&\:\end{array}\right.\end{array}\end{array}$$

where $\:x$ represents the time series, $\:n$ is the length of the series, and $\:{\mathrm{V}\mathrm{a}\mathrm{r}}^{*}\left(S\right)$ is the variance of $\:S$ modified for autocorrelation following Hamed and Rao. Specifically, after removing the trend from the original series, the ranks of the detrended series are used to compute autocorrelation coefficients. Only statistically significant coefficients are retained to compute a correction factor $\:{\mathrm{n}}^{*}$ and the modified variance is $\:{\mathrm{V}\mathrm{a}\mathrm{r}}^{*}\left(S\right)=\mathrm{V}\mathrm{a}\mathrm{r}\left(S\right).{\mathrm{n}}^{*}$, with $\:\mathrm{V}\mathrm{a}\mathrm{r}\left(S\right)=n(n-1)(2n+5)/18$ A positive $\:Z$ value indicates an increasing trend, while a negative $\:Z$ value indicates a decreasing trend.

In this study, we applied the Mann-Kendall test to each grid cell’s time series of the optimal model’s TWSA and its component trend to distinguish spatially consistent, statistically significant trends from random variability. This step is crucial for reliably identifying regions of sustained water storage change and attributing drivers.

## Results and discussion

### Linear trend and significance analysis of TWSA from the three mascon solutions

**Fig. 3:**
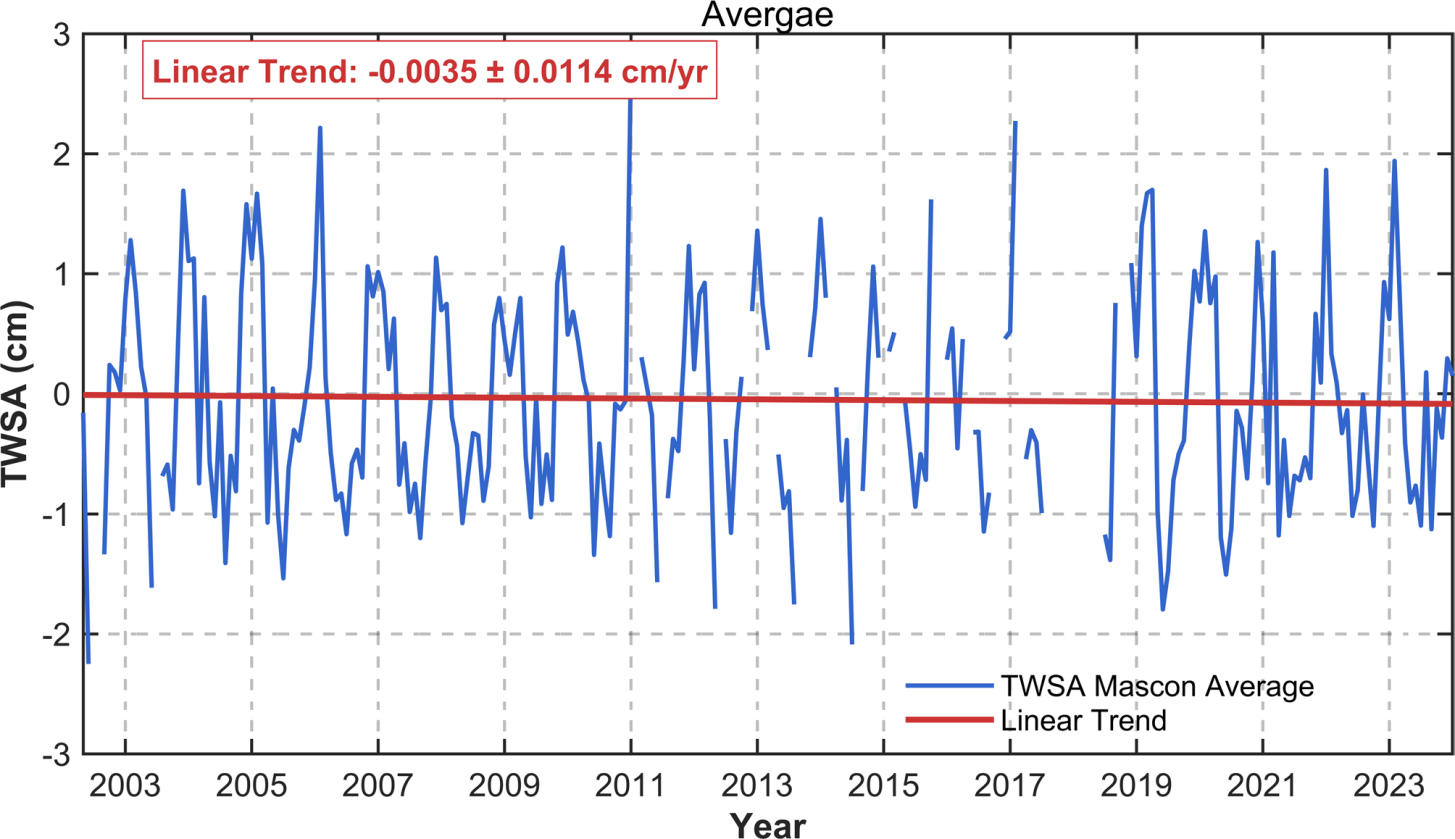
TWSA and linear trend from the average of three Mascon solutions for the period April 2002 to December 2023.

Figure 3 presents the detrended average of the three Mascon solutions and its linear trend, following the same pre-processing procedures as applied to the hydrological models. No interpolation was applied to fill data gaps in the monthly records to preserve the authenticity of the linear trends.

**Fig. 4:**
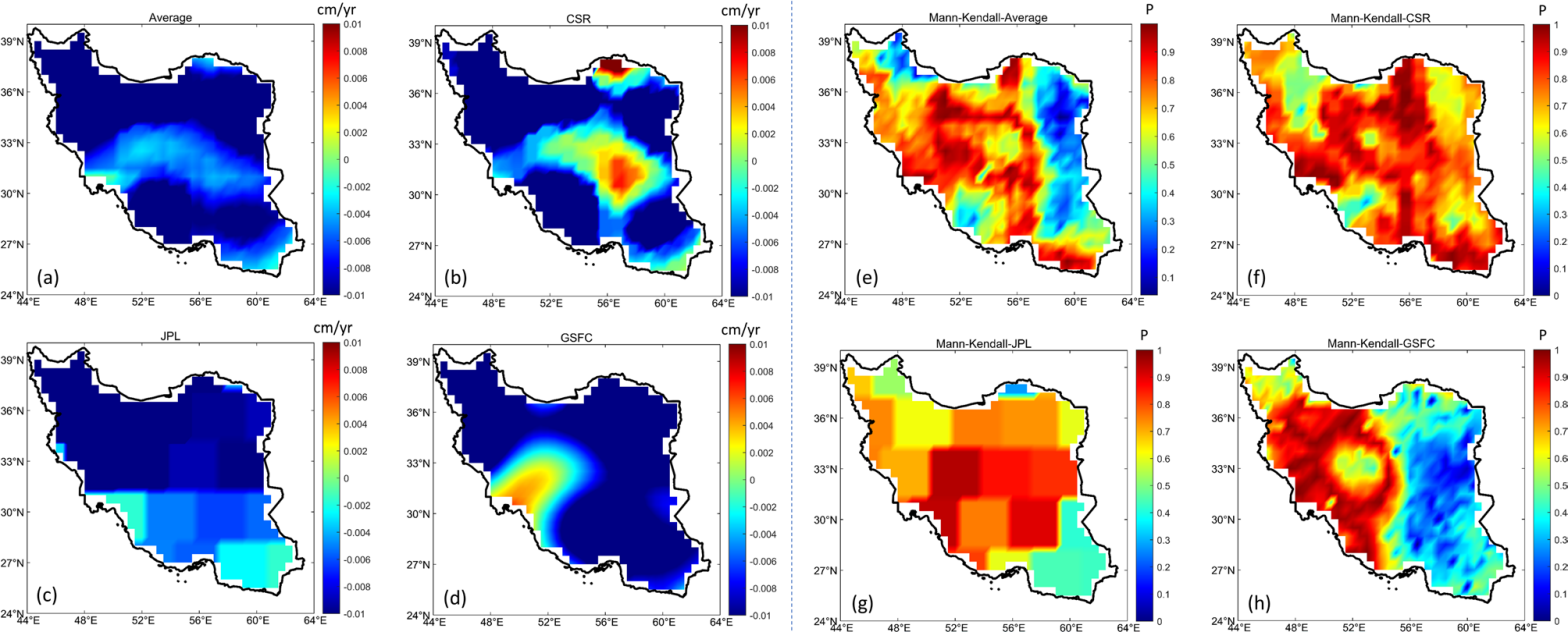
Left (**a**–**d**): Spatial linear trends. (a) Spatial distribution of the linear trend in the averaged TWSA. (**b**–**d**) Spatial distributions of the linear trends in TWSA from the (**b**) CSR, (**c**) JPL, and (**d**) GSFC Mascon solutions, respectively. Right (**e**–**h**): Corresponding Mann-Kendall trend significance test results (Z-scores) for the (**e**) averaged TWSA, (**f**) CSR, (**g**) JPL, and (**h**) GSFC solutions.

Figure 3 presents the time series of TWSA using the average of the three Mascon solutions as a benchmark for hydrological model evaluation. Its linear trend is estimated at − 0.0035 ± 0.0114 cm/yr, which captures the overall direction of water storage change in Iran. However, the associated uncertainty is considerable. This is largely because intensive anthropogenic activities in the region introduce non‑stationary and high‑variance signals into the data. Therefore, to statistically evaluate the significance of linear trends, the Mann‑Kendall trend test was applied in the subsequent spatial analysis over Iran. The results of this spatial significance analysis are presented in Fig. 4.

In the Mann-Kendall trend significance test, a lower p-value indicates higher statistical significance of the trend. Regions with *p* < 0.05 (|Z| > 1.96) are considered statistically significant at the 5% level. A comparison between Fig. 4 (panels (b) and (f), (c) and (g), (d) and (h)) reveals that regions displaying pronounced negative linear trends in TWSA are often associated with relatively high P-values (statistically non-significant). This indicates that high variability in the hydrological time series may be obscuring the trend signal in these areas. Potential explanations include measurement errors, natural climate variability, model imperfections, and the introduction of non-stationary, high-variance signals by anthropogenic activities, all of which can reduce trend significance. A comparison with Fig. 1shows that areas with low statistical significance are predominantly concentrated in the eastern region (44–56°E, 30–40°N), which largely overlaps with densely populated zones. This spatial correspondence indicates that anthropogenic influences are the primary factor contributing to the reduced statistical significance of the observed TWSA trends, a finding consistent with Khaki et al.^37^.

### Preprocessing results of hydrological model components

**Fig. 5:**
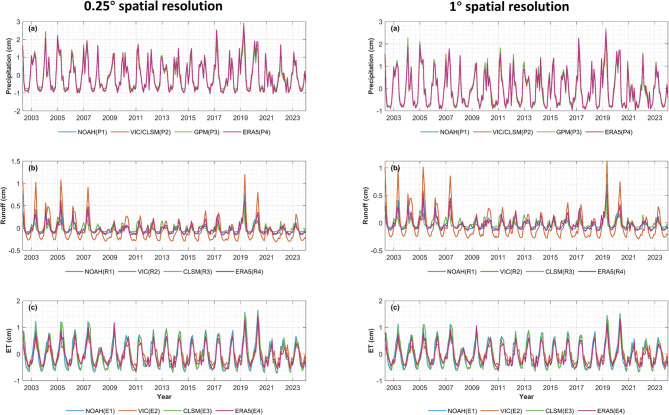
Left: Preprocessed hydrological model components at 0.25° from April 2002 to December 2023: (**a**) precipitation, (**b**) runoff, (**c**) evapotranspiration. Right: Preprocessed hydrological model components at 1° from April 2002 to December 2023: (**d**) precipitation, (**e**) runoff, (**f**) evapotranspiration.

Figure 5 displays the preprocessed components derived from the three GLDAS models and ERA5. These components were spatially harmonized to consistent 0.25° and 1° spatial resolutions using Eqs. (1)–(3) and subsequently aggregated using latitude-weighted averaging. Since GLDAS-VIC and GLDAS-CLSM share the same atmospheric forcing data for precipitation input, the GPM global precipitation data was additionally incorporated as an independent input source for total precipitation.

Figure 5 illustrates the differences among the hydrological models. These inter-model discrepancies reflect the epistemic uncertainties in the parameterization of hydrological processes. Specifically, GLDAS-VIC simulates more pronounced runoff fluctuations, indicating its higher sensitivity to surface runoff generation and moisture stress. GLDAS-CLSM, in contrast, shows greater evapotranspiration variability, which stems from its soil water–groundwater coupling scheme and plant root water uptake parameterization. These differences reflect a basic structural contrast: VIC focuses on runoff generation^38^, whereas CLSM emphasizes groundwater-mediated terrestrial–atmosphere interactions^39^.

### Construction and validation of multi-model combinations

**Fig. 6:**
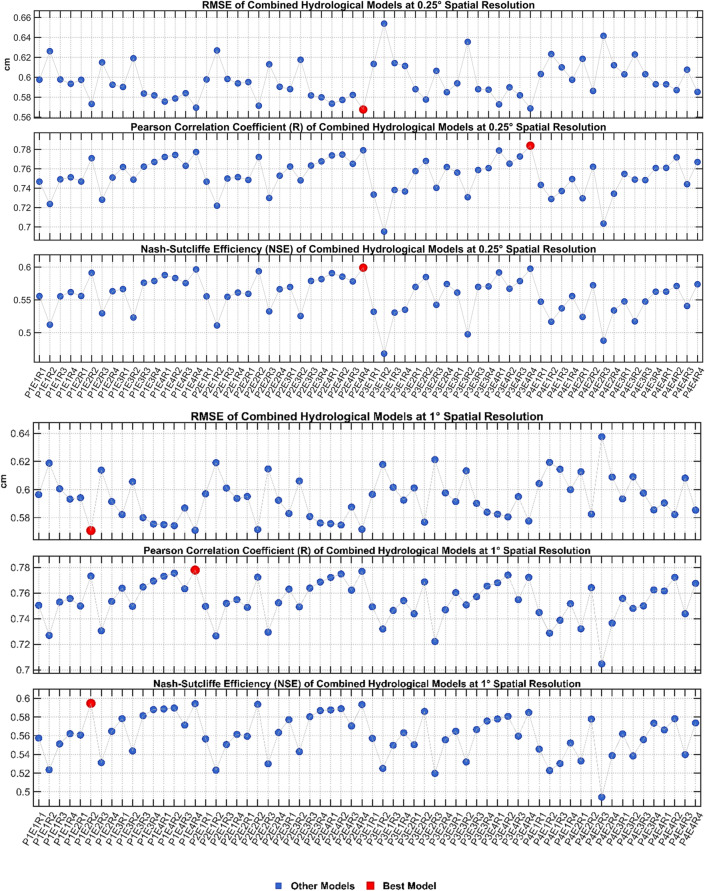
RMSE, R, and NSE between the combined hydrological models and the average Mascon TWSA.

Based on the data presented in Fig. 5, the four precipitation datasets (designated as P1: Noah, P2: VIC/CLSM, P3: GPM, P4: ERA5), four evapotranspiration datasets (designated as E1: Noah, E2: VIC, E3: CLSM, E4: ERA5), and four runoff datasets (designated as R1: Noah, R2: VIC, R3: CLSM, R4: ERA5) were first aligned by removing months with missing data in the corresponding Mascon records. Using Eq. (4), these datasets were systematically combined to generate 64 distinct hydrological model combinations at two different spatial resolutions. The RMSE, R, and NSE were then calculated between each combined TWSA time series and the benchmark Mascon mean. The results are shown in Fig. 6.

**Fig. 7:**
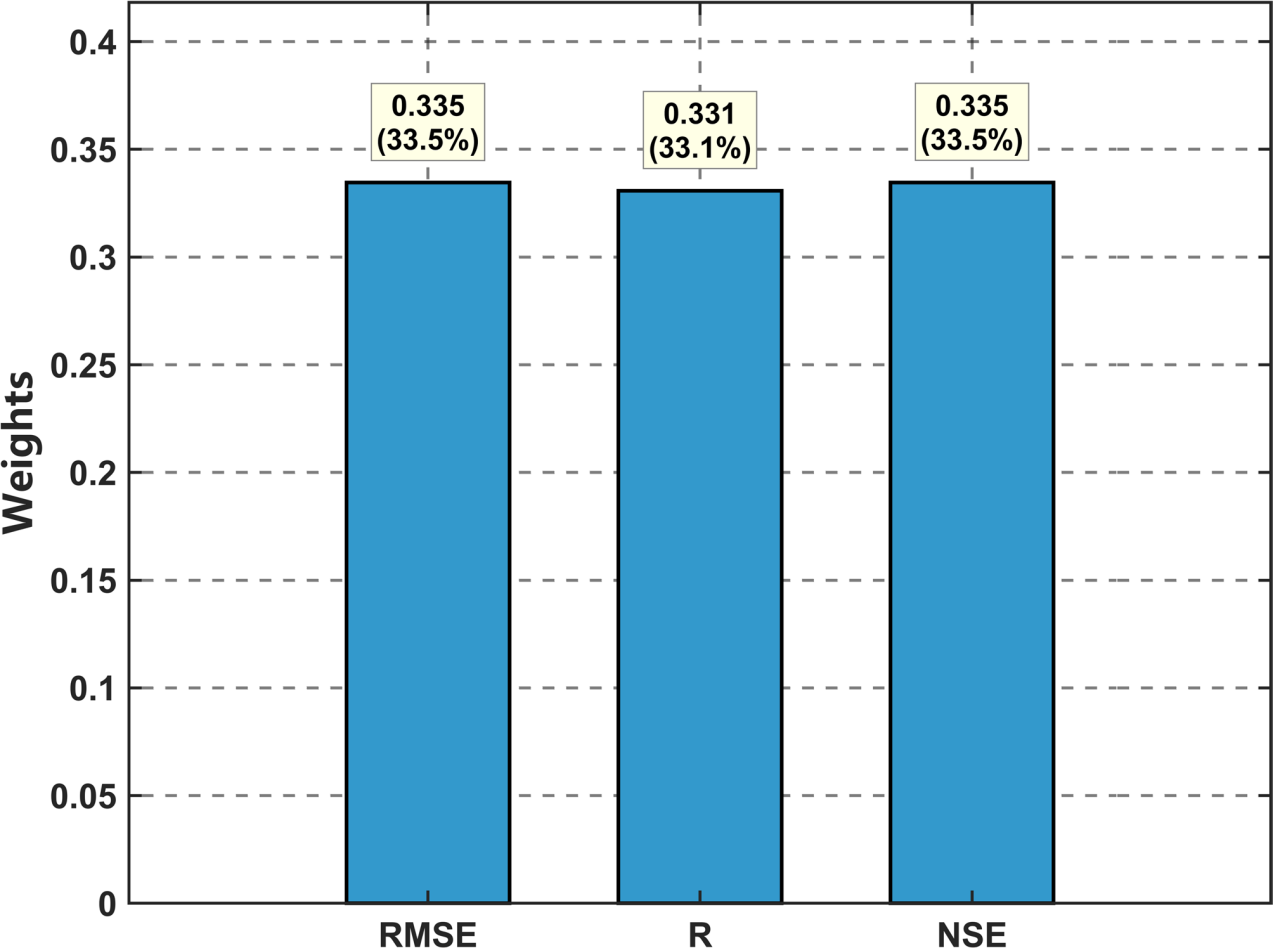
Assigned weights for the three evaluation metrics based on PCA.

Analysis of Fig. 6 begins by defining the three key metrics employed for model evaluation. Root Mean Square Error (RMSE, ranging from 0 to +∞) represents the absolute magnitude of error, with lower values indicating superior performance. The correlation coefficient (R, ranging from − 1 to 1) measures linear agreement, where values closer to 1 signify a stronger relationship. Nash–Sutcliffe Efficiency (NSE, ranging from –∞ to 1) assesses the model’s predictive skill relative to the observed mean, with values approaching 1 denoting better performance. After converting RMSE to a positive indicator and normalizing all three metrics, PCA was performed. PC1 explained 97.7% of the total variance, indicating strong agreement among the three metrics. Therefore, model rankings are robust to the choice of weighting scheme. The absolute loadings of PC1 were normalized to sum to unity to derive the weight for each metric, and the resulting weights are shown in Fig. 7.

**Fig. 8:**
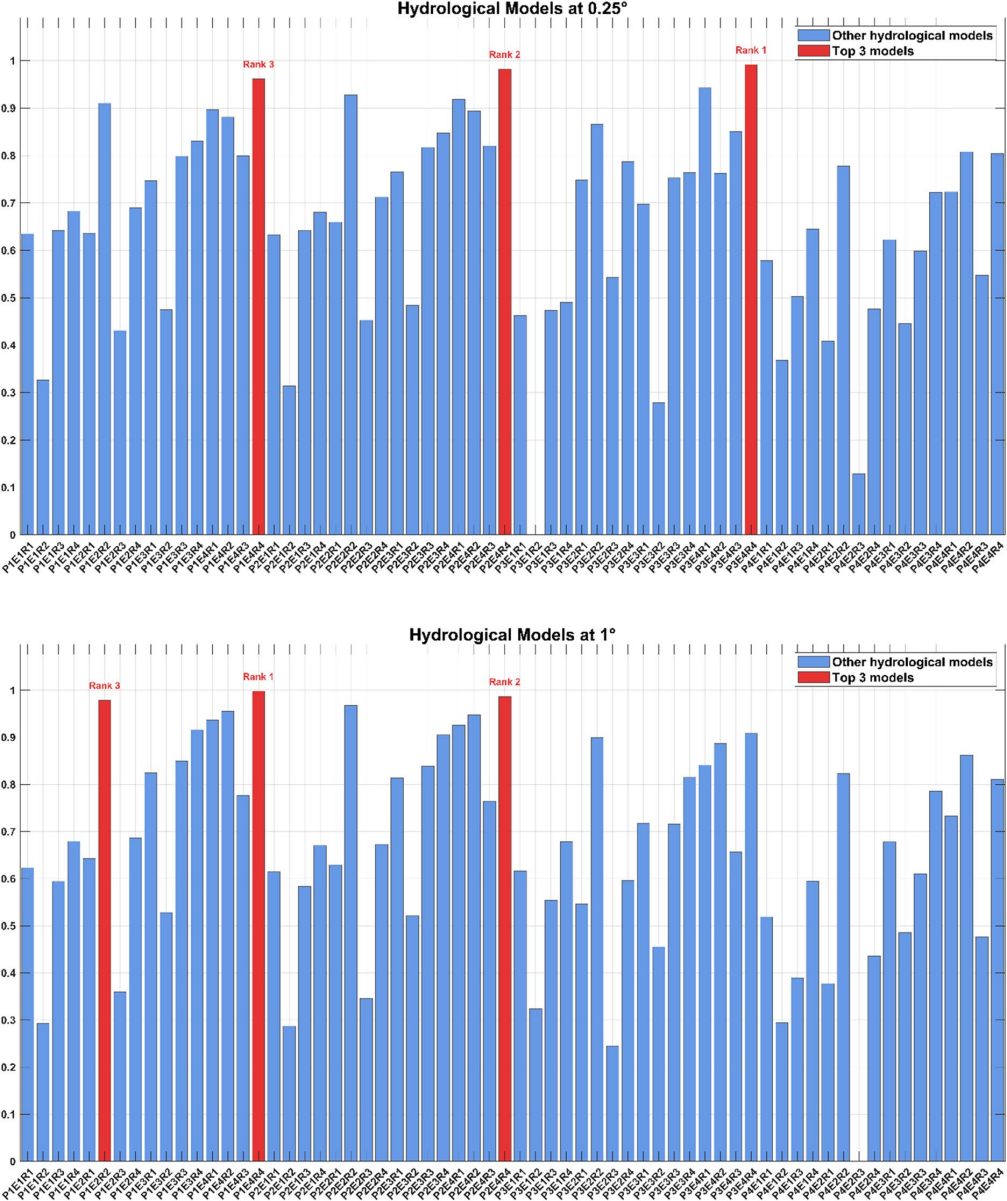
Composite scores of the hydrological model combinations at 0.25° and 1° spatial resolutions.

From Fig. 7, the weights assigned to the three evaluation metrics are 0.335, 0.331, and 0.335, respectively. Using Eq. (9) as the weighted sum of the normalized metric values, a composite score was calculated for each hydrological model combination. Models with higher composite scores were considered to exhibit better overall performance. The composite scores for the hydrological model combinations at both spatial resolutions are presented in Fig. 8.

**Fig. 9:**
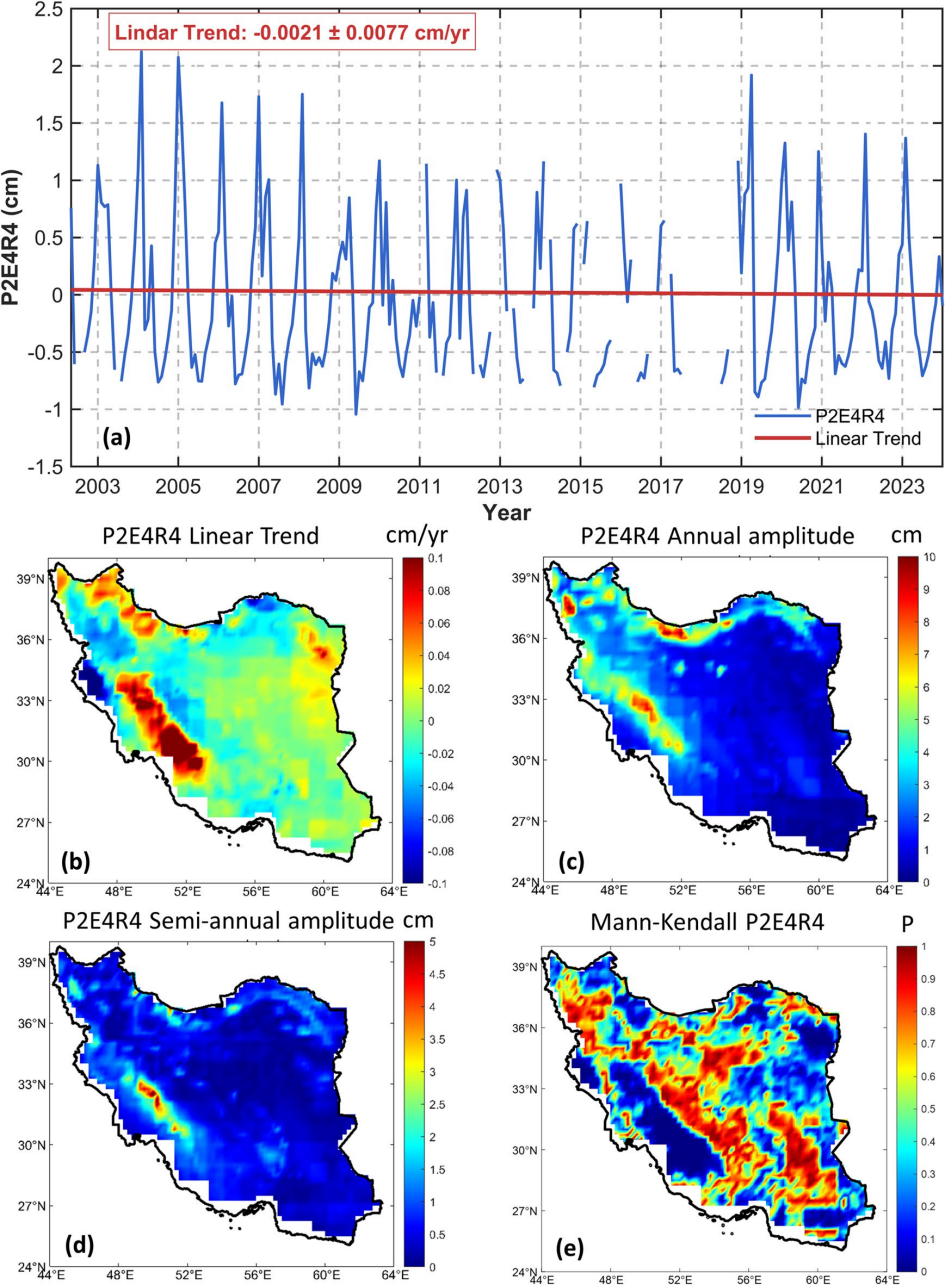
(**a**) Linear trend of the combined model P2E4R4 (P2: CLSM/VIC, R4: ERA5, E4: ERA5); (**b**) Spatial linear trend of P2E4R4; (**c**) Annual amplitude of P2E4R4; (**d**) Semi-annual amplitude of P2E4R4; (**e**) Mann–Kendall trend significance test results for P2E4R4.

At the 0.25° spatial resolution, the hydrological model outputs were generated using nearest-neighbor replication, which introduces artificial spatial details without adding new information. In contrast, the 1° spatial resolution fails to adequately represent the hydrological conditions over Iran. As shown in Fig. 8, the combinations P3E4R4, P2E4R4, and P1E4R4 perform best at 0.25° resolution, while P2E4R4, P1E4R4, and P1E2R2 exhibit superior performance at 1° resolution. Based on a comprehensive evaluation, the P2E4R4 combination—which exhibited the lowest RMSE along with the highest NSE and high R—was selected as the optimal hydrological model configuration for subsequent trend analysis. However, this selection should be interpreted with caution: statistical metrics do not guarantee performance under all conditions, equifinality may exist among parameter sets, and non‑stationary signals (e.g., land use change, groundwater extraction) could reduce its robustness over time^40^. The spatial trends and Mann‑Kendall trend test of TWSA derived from this optimal combination are presented in Fig. 9.

**Fig. 10:**
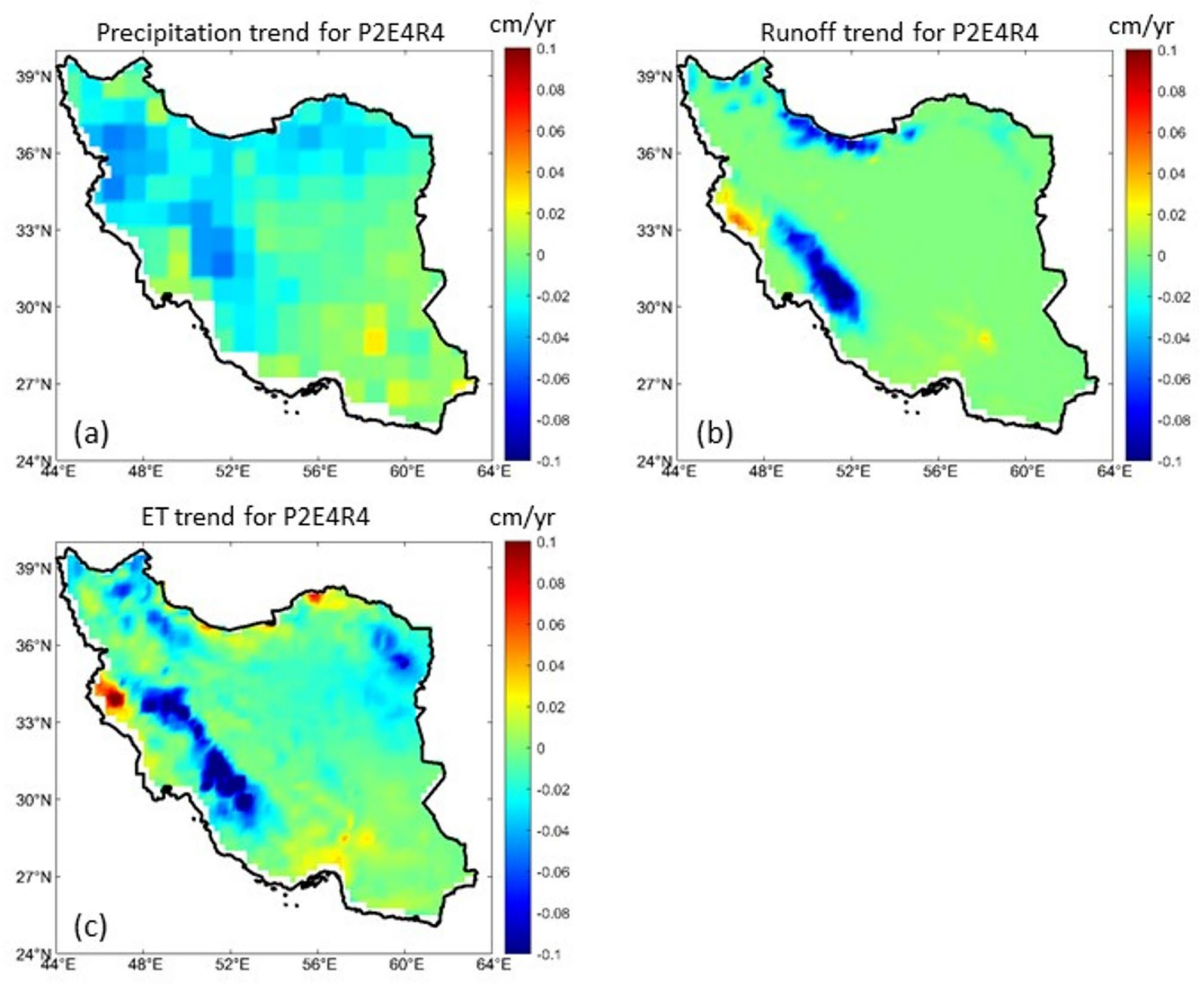
Components of the combined hydrological model: (**a**) Precipitation, (**b**) Runoff, and (**c**) ET.

As shown in Fig. 9a, the linear trend of the optimal combined model P2E4R4 is − 0.0021 ± 0.0077 cm/yr, which is lower than that of the Mascon data, due to the exclusion of anthropogenic influences in the hydrological models. In reality, anthropogenic water depletion in Iran is severe. Naderi et al^41^. reported that in 2021, anthropogenic groundwater extraction accounted for approximately 60% of the total water supply in Iran, with agricultural, municipal, and industrial uses comprising about 92%, 7%, and 1%, respectively. Figure 9b and e present the spatial analysis over Iran, where a larger annual amplitude indicates a stronger response to the seasonal cycle, and a larger semi-annual amplitude suggests stronger system nonlinearity or multi-frequency forcing. Notably, the eastern region (33–36°N, 58–62°E) also exhibits a pronounced negative trend, but with relatively small annual and semi-annual amplitudes, implying a relatively steady decline dominated by background factors. The Mann–Kendall trend test results shown in Fig. 9e reflect the statistical significance of natural water storage changes. Notably, this significance is also jointly affected by excessive groundwater extraction and model errors. Large amounts of extracted water are used for agricultural irrigation; a portion of this extracted water recharges groundwater sources, while the remainder is lost through evapotranspiration and runoff. These processes introduce non‑stationary, high‑variance signals^42^, leading to significantly higher p‑values in the Mann–Kendall trend test. The spatial trends of the key hydrological components simulated by the combined model are illustrated in Fig. 10.

According to Fig. 10, the TWSA decline in northern (36–40°N, 44–52°E) and southwestern (28–34°N, 48–54°E) Iran is predominantly driven by runoff and evapotranspiration, where their contributions outweigh the effect of precipitation increases. In the eastern region (33–36°N, 58–62°E), the TWSA decline is primarily driven by evapotranspiration. This aligns with the findings of Forootan et al.^43^, who also identified that meteorological factors are key contributors to negative water flux trends across several Iranian basins. In the western region (33–36°N, 46–48°E), a less pronounced TWSA decline is governed by the combined effects of evapotranspiration and runoff. In other regions, TWSA changes are primarily determined by the interplay between precipitation and evapotranspiration, with minimal net change resulting from their opposing effects largely canceling each other out.

### Anthropogenic contribution to TWSA

**Fig. 11:**
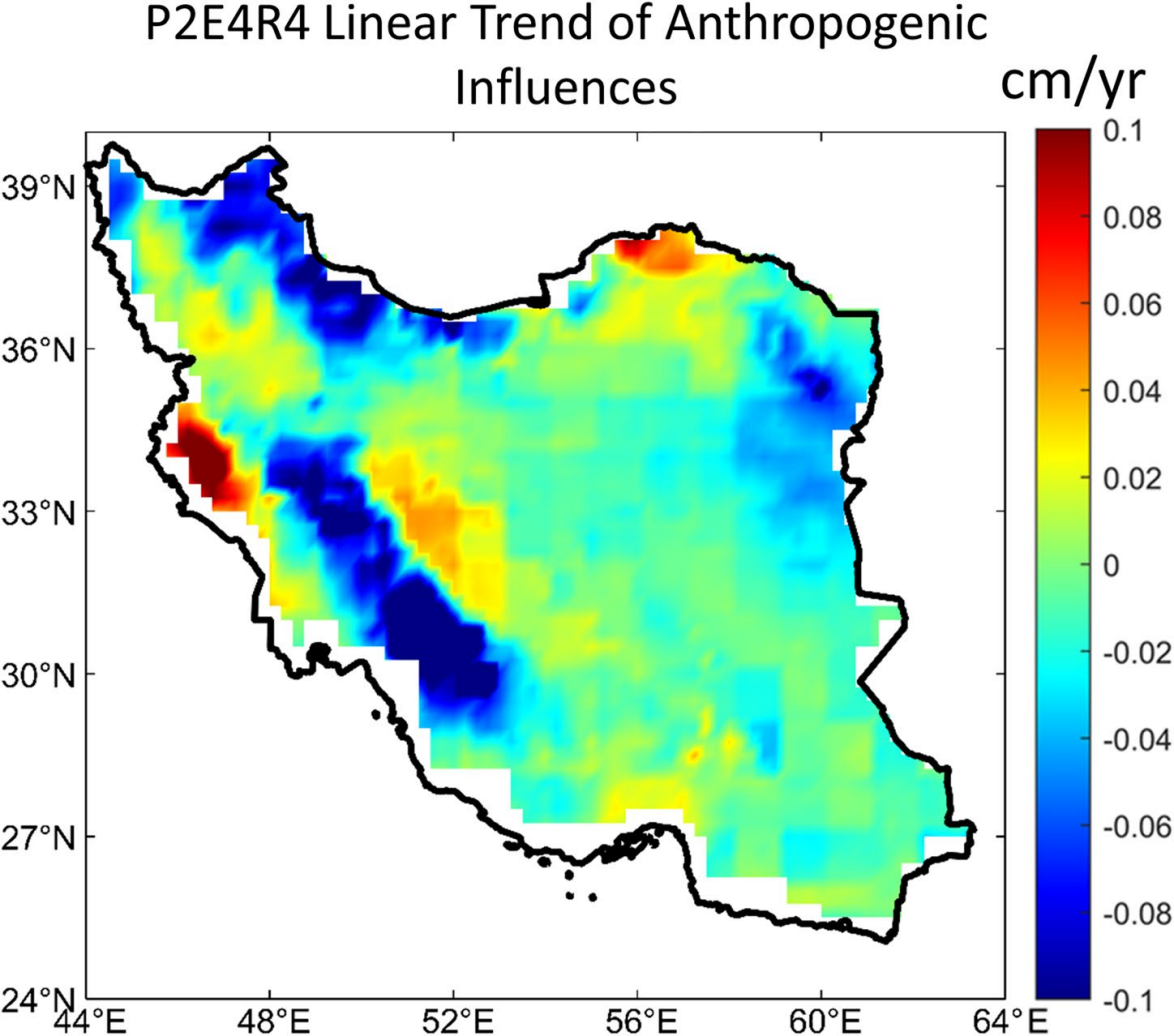
P2E4R4 Linear trend of anthropogenic influence.

The TWSA derived from Mascon data represents changes in terrestrial water storage anomalies, while the TWSA obtained from selected hydrological models represents changes attributable to natural hydrological processes. Given the inherent uncertainties among models, the difference between these two time series, calculated based on the water balance equation, represents the combined effect of anthropogenic influences and model errors. The results are presented in Fig. 11.

As shown in Fig. 11, pronounced negative trends are observed in the northern (36–40°N, 44–52°E) and southwestern (28–34°N, 48–54°E) regions, which correspond to densely populated areas (Fig. 1). In contrast, the central region (27–36°N, 54–58°E) exhibits a stable to slightly increasing anthropogenic TWSA trend, where the population density is also lower.

### Limitations

**Fig. 12:**
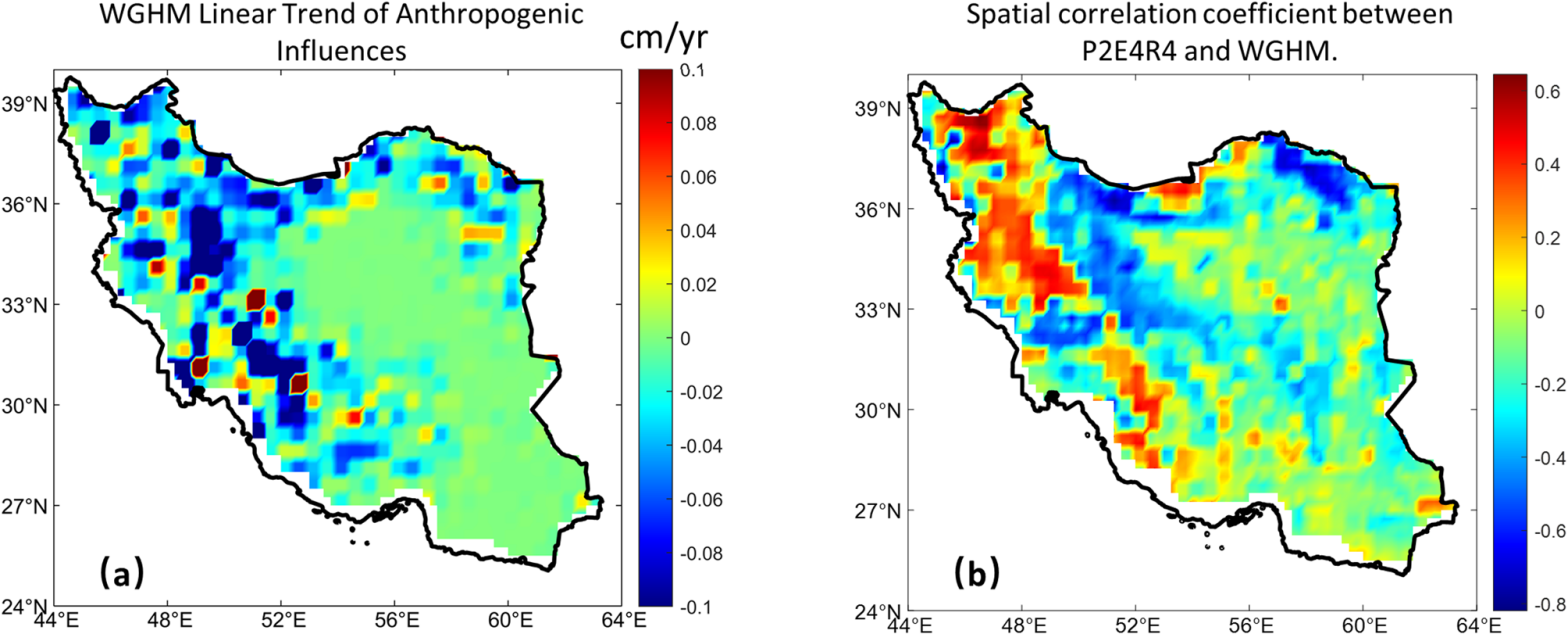
WGHM Linear trend of Anthropogenic influence and its spatial correlation with P2E4R4.

The method adopted in this paper mainly assesses the relative performance of different datasets. Consequently, the anthropogenic influence signal obtained in Fig. 11 is not an exact quantitative result. This conclusion incorporates both the impacts of anthropogenic water use and the discrepancies between various hydrological models. To further validate the discrepancies between models, the anthropogenic water use outputs from the WGHM hydrological model are used to compare with the results in Fig. 11 for validation, and the spatial correlation is computed. The results are shown in Fig. 12.

Figure 12 shows that the anthropogenic water use impact areas from P2E4R4 and WGHM largely correspond. However, some regions exhibit negative correlations; for example, in the northern area (36–38°N, 58–62°E), the negative correlation exceeds 0.6 in absolute value. This is caused by model errors between different hydrological models and Mascon^44^. Future studies should introduce external data, such as in-situ groundwater level measurements, reservoir storage records, and irrigation water use statistics, to correct these model errors. Furthermore, Iran was selected as a case study to evaluate the explanatory power of a natural-water-cycle‑dominant framework (ΔP – ΔET – ΔR) for terrestrial water storage changes under significant anthropogenic forcing, prior to reassessing the human contribution based on the combined hydrological models. Consequently, in a region like Iran with intense human disturbance, model performance metrics (RMSE, R, and NSE) between the hydrological models and GRACE data are inherently challenged. Conversely, in regions with negligible human impact, the combined‑model TWSA provides a viable alternative for filling GRACE data gaps, as the methodology would be expected to yield a substantially better fit under such conditions.

## Conclusions

1. This study systematically evaluated 128 hydrological model combinations to identify an optimal configuration for reconstructing anthropogenic influences on TWSA across Iran. PC1 explained 97.7% of the variance among RMSE, R, and NSE, confirming that model rankings are robust to weighting choices. The P2E4R4 combination, integrating multi-source independent datasets, showed the best agreement with Mascon data, highlighting the value of combining diverse data to reduce uncertainties and establish a reliable benchmark.
2. Spatial analysis revealed significant heterogeneity in TWSA decline across Iran, with the northern (36–40°N, 44–52°E) and southwestern (28–34°N, 48–54°E) regions identified as two core depletion zones characterized by distinct seasonal and sub-seasonal variability. These differences reflect the varying dominance of climatic and anthropogenic processes across regions. Attribution analysis further indicated that increases in runoff and evapotranspiration are the primary drivers of water storage loss in the main depletion zones, while in the eastern region, evapotranspiration plays a dominant role.
3. The Mann–Kendall trend significance test results for both the Mascon data and the combined hydrological model exhibit relatively high p-values, which are attributable to the combined effects of anthropogenic water use, measurement errors, natural climate variability, and model uncertainties. For instance, intensive human activities (e.g., groundwater overexploitation) may introduce non‑stationary, high‑variance signals into hydrological time series, thereby masking the statistical detectability of underlying trends. Furthermore, model errors among the combined hydrological models also affect the statistical significance.

## Data Availability

The GRACE and GRACE-FO Mascon data used in this study are openly available from the Jet Propulsion Laboratory (JPL) at https://grace.jpl.nasa.gov/data/get-data/jpl_global_mascons/, the Center for Space Research (CSR) at https://www2.csr.utexas.edu/grace/RL06_mascons.html, and the Goddard Space Flight Center (GSFC) at https://earth.gsfc.nasa.gov/geo/data/grace-mascons (all accessed on 20 August 2024). The hydrological model data were obtained from the following sources: GLDAS data were downloaded from the NASA Earthdata Search portal (https://search.earthdata.nasa.gov/search? p=!C2491772131-POCLOUD&q=gldas, accessed on 20 August 2024); ERA5 data were sourced from the Copernicus Climate Data Store (https://cds.climate.copernicus.eu/datasets, accessed on 20 August 2024); and WGHM data are available from the PANGAEA repository (https://doi.pangaea.de/10.1594/PANGAEA.948461, accessed on 20 August 2024).
